# Overcoming the oral delivery challenges of venetoclax: advancing a clinically improved Bcl-2 inhibitor

**DOI:** 10.1007/s13346-026-02093-x

**Published:** 2026-03-10

**Authors:** Yukta Hegishte, Paul Joyce, Clive Prestidge, Kristen Bremmell

**Affiliations:** School of Pharmacy and Biomedical Science, Adelaide University, Adelaide, South Australia 5000 Australia

**Keywords:** Venetoclax, Pharmacokinetics, Bioavailability, Delivery, Solubility, Formulations, Lipid-based formulations

## Abstract

**Abstract:**

The Bcl-2 inhibitor, Venetoclax, is a notable example of a therapeutic emerging from modern drug development pipelines with structural and physiochemical properties sitting beyond Lipinski’s “Rule of 5”. The classification of VTX as a biopharmaceutical classification system (BCS) Class IV compound aligns with its observed low solubility and permeability in the gastrointestinal tract. Its inherent “brick-dust” properties further limit solubility in lipid delivery vehicles, collectively constraining its maximum potential for oral absorption. With a low fasted oral bioavailability (< 5%) and a fivefold positive food effect, a clinical dose of 600 mg is required daily and is prescribed to patients with a meal to ensure therapeutic efficacy. The impact of critical formulation design parameters on the performance of previously reported formulation strategies such as amorphous solid dispersions, nanocrystals and lipid-based systems will be examined. Mechanistic insight into how bio-enabling strategies improve oral absorption of VTX is provided, including enhanced solubility in lipid vehicles, improved solubilisation upon dispersion, mitigation of the gastrointestinal pH gradient, reduced particle size, amorphous state enhanced-solubility and reduced metabolism. Potential alternative formulation strategies such as inorganic vehicles or advanced lipid-based systems are reviewed, with their capability to enhance VTX’s bioavailability whilst reducing the high clinical dose and dependence on food for optimal absorption. Key silica and polymer selection is necessary for loading VTX mesoporous silica nanoparticles and polymeric nanoparticles. Novel formulation avenues, including solidified self-emulsifying systems, supersaturated self-emulsifying systems, solid-lipid nanoparticles, and nanostructured lipid-carriers, are also explored. Systematic in vitro and in vivo investigations of these formulations can provide essential in vitro-in-vivo correlation insights. Integration of lipid formulation-specific parameters into a physiologically based pharmacokinetics model can facilitate improved clinical translation of lipid-based VTX formulations.

**Article highlights:**

- Venetoclax can benefit from reformulation using novel bio-enabling strategies for enhanced oral absorption
- Rational formulation design can beneficially affect the performance of bio-enabling systems
- Use of advanced lipid-based formulations and/or inorganic vehicles, can enhance pharmacokinetics and reduce limitations of the current clinical use of venetoclax
- Combining advanced formulation science and modelling is key for clinical success of venetoclax-based lipid formulations

**Graphical Abstract:**

**Figure:**
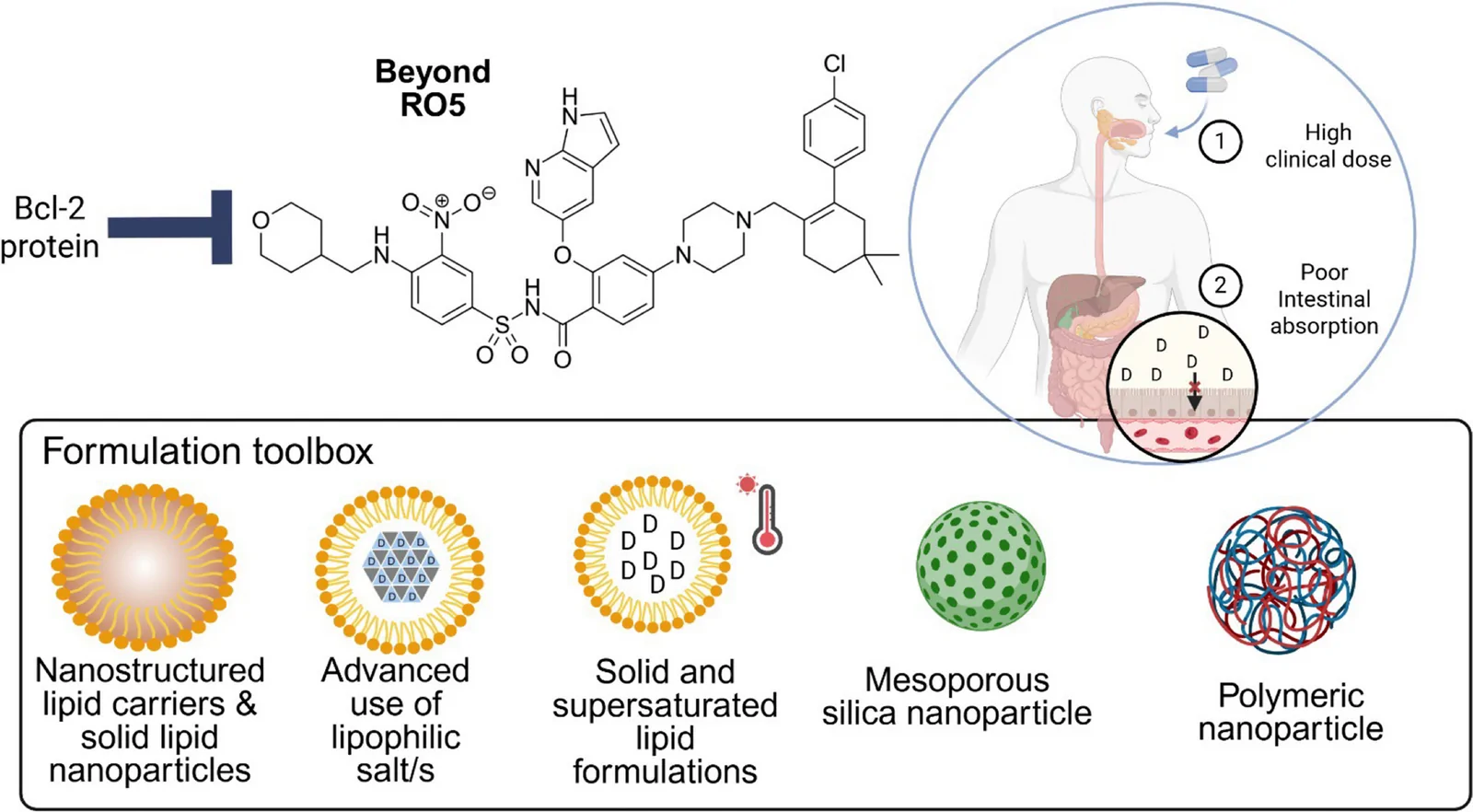

## The venetoclax story

Since the 1970’s, standard-of-care for haematological malignancies consisted of a remission induction phase using cytarabine and an anthracycline, followed by a consolidation phase consisting of higher doses of cytarabine with or without blood-forming stem cell transplantation [1]. This non-selective nature of broad-spectrum chemotherapy was unable to target multiple genetic aberrations, hence resulting in unsatisfactory prognosis and relapse after remission [1]. A landmark discovery on the role of an anti-apoptotic protein i.e. B-cell lymphoma (Bcl-2) and its pivotal role in cancer development is regarded as a revolution in the study of oncogenes [2]. Overexpression of Bcl-2 proteins in cancerous cells resisted cell death, leading to sustained growth of various leukemias and lymphomas. Thus, anti-apoptotic Bcl-2 proteins were recognised as compelling targets for therapeutic intervention and led to a positive evolution in the treatment landscape [2]. A collaboration between the Walter Eliza and Hall Institute and two biopharmaceutical companies, Genentech and AbbVie Inc. initiated a long 25-year drug discovery campaign which resulted in the regulatory approval of a first-in-class potent, B-cell lymphoma 2 (Bcl-2) inhibitor, Venetoclax (VTX) in 2016 [2]. Binding of VTX to the correct domain of the Bcl-2 protein activates pro-apoptotic proteins that further active the intrinsic apoptosis pathway and lead to mitochondrial outer membrane permeabilization (MOMP) [3, 4]. MOMP releases cytochrome C into the cytoplasm, ultimately leading to cellular apoptosis. Selective binding of VTX antagonizes Bcl-2’s anti-apoptotic action, and this highly selective action renders the benefit of VTX over traditional chemotherapy-based cytotoxic drugs as the function of Bcl-2 is non-essential in many normal cells but overexpressed in malignant cells, reducing off-target toxicity [4].

VTX discovery was based on a screened campaign of 100,000 for their affinity, selectively, and efficacy towards Bcl-2 proteins. Compound ABT-737 was identified, with high affinity to the Bcl protein family and anti-tumour activity in preclinical models and patient samples [5]. A patent application containing pharmacological data on the Bcl-2 binding inhibition constants and cell viability assays of 404 compounds was filed in May 2010 [6]. Due to heavy medicinal chemistry approaches, ABT-737 displayed unfavourable physicochemical properties for clinical translation, thus further structural modifications led to the discovery of ABT-263 (navitoclax) [5]. Navitoclax displayed a pharmacokinetic profile and antitumour activity in clinical studies akin to ABT-737. However, the clinical translation in this scenario was impacted by its co-inhibition of another Bcl protein, Bcl-X_L_. As Bcl-X_L_ is critical for platelets survival, patient given navitoclax suffered extensive haemorrhage [5]. Therefore, further structural refinement was required, culminating in ABT-199 (VTX), a selective Bcl-2 inhibitor with a 200-fold reduction in binding to Bcl-X_L_, rendering it suitable for clinical translation [5].

Structure-based discovery yielded VTX, a compound characterized by a large molecular weight of 868 g/mol, extensive hydrophobicity, low solubility of < 0.0042 µg/mL in aqueous media and a logP value of 8.1 [7]. Such challenging physiochemical properties result in solubility-limited dissolution (dissolution rate limited by the intrinsic solubility of VTX) and low human effective permeability rates (P_eff_) across intestinal membranes of 0. 49 × 10^–4^ cm/s, restricting absorption and classifying VTX as a biopharmaceutical classification system (BCS) Class IV candidate [7]. VTX is innovatively formulated by AbbVie Inc. as an amorphous solid dispersion (ASD) and marketed as Venclexta^®^, which is globally approved for chronic lymphocytic leukemia (CLL) or small lymphocytic lymphoma (SLL), either as a monotherapy or in combination with obinutuzumab and rituximab [2, 8]. Venclexta^®^ consists of VTX in an amorphous state as opposed to a crystalline state, facilitating enhanced solubility. Amorphous solids exhibit short-range order as opposed to long-range order of crystalline materials, leading to increased solubility and dissolution profiles. Risks of phase instability of an amorphous state in the gastro-intestinal tract can remove these associated benefits. To address this, the formulation stabilises the amorphous drug with povidone i.e. a synthetic water-soluble polymer to mitigate the risk of drug re-crystallisation. This yields kinetically stable amorphous solid dispersions (ASDs) with no crystallisation being reported for up to 6 months [9]. Nonetheless, Venclexta^®^ fails to display desired oral bioavailability and instils several limitations to patient use, drug toxicity and treatment efficacy. The absolute oral bioavailability of Venclexta^®^ was estimated to be only 5.4% under fasting conditions [10]. A high clinical dose of 600 mg and a large quantity of polymer as a formulation excipient limits tablets to a loading of 100 mg, a high tablet weight and size, and ultimately pill burden when patients are required to take up to four or six tablets as per their regimen [10]. A high pill burden negatively impacts on patient compliance and adherence, increases the risk of formulation induced adverse effects, and induces concerns for elderly patients who may experience difficulties in swallowing large tablets [11]. Exposure of VTX is associated with cardiotoxicity, neutropenia, gastrointestinal side effects and with growing use, emerging drug resistance mechanisms pose a concern for its long-term efficacy [9, 12, 13].

VTX’s selective and potent nature as a Bcl-2 inhibitor has extended its clinical use, with downstream approvals as a combination therapy with hypomethylating agents (azacytidine or decitabine) or low-dose cytarabine in acute myeloid leukemia (AML) and a type II anti-CD20 antibody (obinutuzumab) for CLL [2, 8]. Various clinical trials have demonstrated clinical efficacy in other types of haematological malignancies including mantle cell lymphoma, diffuse large B-cell lymphoma, follicular lymphoma, acute lymphoblastic leukemia and multiple myeloma, however the drug remains investigational for such indications pending approval [14]. Breakthrough preclinical findings of a collaboration study between Walter Eliza and Hall Institute and Doherty Institute revealed the cytotoxic effect of VTX on human immunodeficiency (HIV) virus-infected cells and delayed virus re-emergence of the virus in humanized mice [15]. Such findings are the basis of an upcoming Phase I/IIb clinical trials due to investigate the repurposing of VTX for HIV treatment [15]. This instils a requirement for ongoing optimization of VTX delivery by optimisation of bioavailability in the fasted state compared to the commercial formulation, to enable dose reduction and reduced variability in drug exposure [16]. Recently, Nguyen et al. co-encapsulated VTX alongside a kinase inhibitor in liposomes, elucidating the benefit of nanocarrier platforms for enhanced efficacy against AML [17]. Liposomes remain largely unsuitable for oral delivery due to their inherent instability in the GIT, limiting their use for intravenous delivery.

This review provides a comprehensive analysis of the physical, structural, physiochemical and stability characteristics of VTX, to have a thorough understanding of the biopharmaceutical properties necessary for effective oral formulation and optimal performance. An overview of trialled formulation/s for oral delivery encompassing both non-nanocarrier and nanocarrier strategies designed to address the limitations of the commercial formulation is provided, focusing on the role of formulation factors in drug delivery. This will set the foundation to discuss the use of alternative approaches and potential enhancements to existing formulation strategies which are yet to be applied to VTX, with the potential that use of these alternative strategies can provide improved pharmacokinetic parameters. Integration of in silico tools for screening of excipients and physiologically based pharmacokinetics modelling (PBPK) is discussed as powerful aids in formulation development and clinical translation. A critical review of the history, current and improved bio-enabling formulations for the enhanced performance of VTX is of interest and use to formulators in academia and industry, as well as clinicians, equipping them with the insight on recent progress and opportunities to advance the field of oral delivery of VTX.

## Design considerations for the development of improved oral formulations of VTX

### Structural, physicochemical and physical properties of VTX

**Fig. 1:**
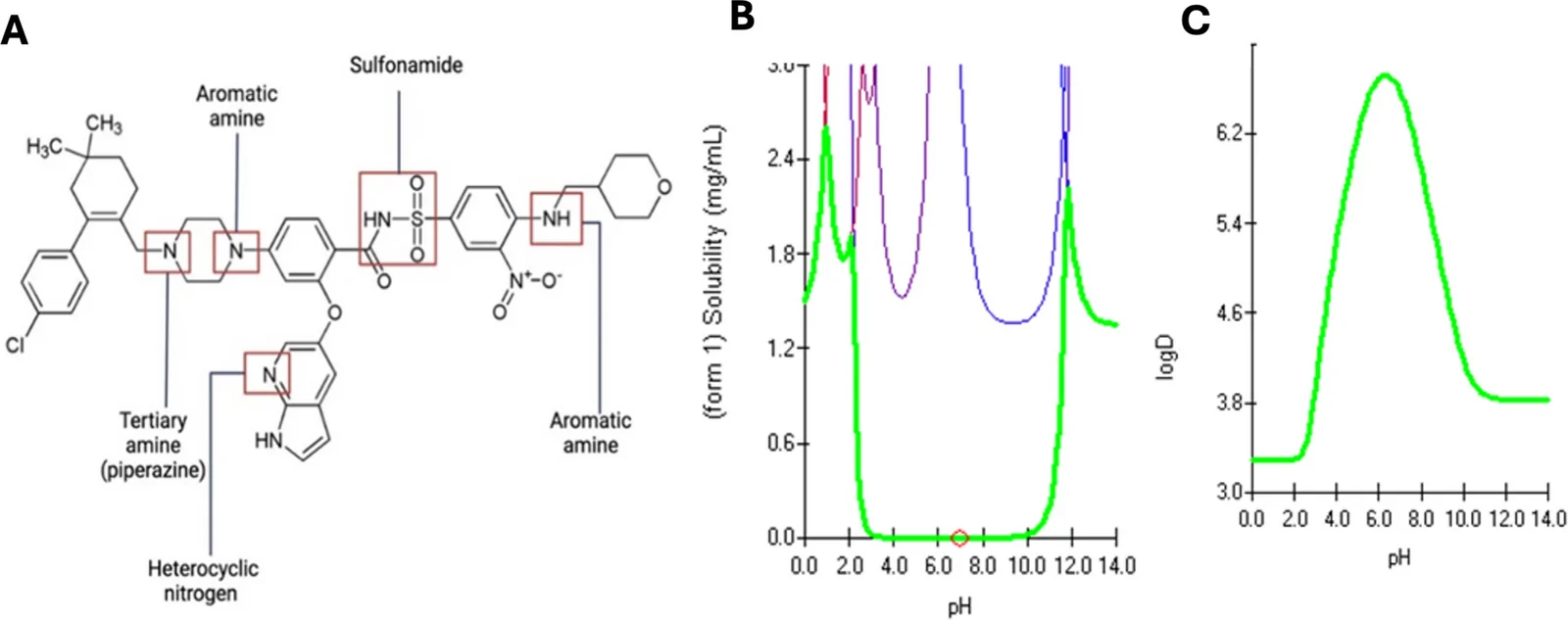
**A)** Chemical structure of VTX with functional groups highlighted **B)** pH solubility profile extracted from Gastroplus program of form 1 (VTX) where coloured lines indicate the specific solubility of individual pKa values in relation to pH and green line indicates solubility of VTX as a function of all pKa values in relation to pH **C)** logD profile of VTX as a function of pH

VTX is a dark yellow non-hygroscopic amorphous glass-former exhibiting an onset melting point of 138 °C. Belonging to the class of pyrrolopyridines, VTX contains a five-membered pyrrole ring fused to a six-membered pyridine ring. Alongside this, VTX contains multiple ionisable groups, with the pKa values of sulfonamide and piperazine groups physiologically relevant (refer to Fig. 1A). VTX is characterized by a relatively high molecular weight (868.4 g/mol) and high hydrophobicity, evidenced by a log P value of 8.1 [18]. VTX exhibits pH-dependent solubility with minor solubility at pH 1 and 12.9, however practically insoluble from pH 4 to 7.4 (refer to Fig. 1B). A slight increase in solubility is observed at highly basic conditions between pH 12–14. Literature reports a solubility value of 0.0023 mg/ml at pH 1, further decreasing to 0.0004 mg/mL at pH 7.4 [10]. Regulatory data similarly describe higher solubility at pH 1 and pH 12, with lower solubility at pH 4 and 7 [12]. This is consistent with the trend observed in Fig. 1B. The pH-solubility trend also inversely correlates with the logD profile, whereby higher lipophilicity is associated with reduced solubility and vice-versa. A high logD value of 6.5 (Fig. 1C) supports its highly lipophilic structure being liable to poor aqueous solubility, high first-pass metabolism and high binding to plasma proteins/tissues [19]. VTX displays biophysical properties of a “brick-dust” compound, evidenced by a maximum, low solubility of 19.4 mg/mL across a range of long-chain and medium-chain lipid excipients [20]. Polymorphism can heavily influence the biopharmaceutical properties of the isolated product, whereby a patent by Rao et al. disclosed the polymorphic behaviour of VTX by detailing the preparation processes of 22 different crystalline forms [21]. Glass-forming properties of VTX were investigated by Koehl et al. who observed melting during the first heating cycle, formation of amorphous VTX with absence of crystals during a cooling cycle and no recrystallization during the reheating cycle, categorising VTX as a class III glass former [20]. Table 1 summarizes the key physiochemical properties of VTX, including its biorelevant solubility values in water, fasted-state intestinal media (FaSSIF) and fed-state intestinal media (FeSSIF).

**Table 1:** Physico-chemical characteristics of VTX [7, 18]

Physiochemical property	VTX Properties
Molecular formula	C_45_H_50_CIN_7_O_7_S
Molecular weight (g/mol)	868.4
LogP	8.1
pKa	3.4 (sulfonamide group) and 10.3 (piperazine group)
Permeability (cm/s)	Low to moderate (4.9 × 10^–5^)
Melting point (°C)	138
Intrinsic water solubility (µg/mL)	0.0038
FaSSIF solubility (µg/mL)	5.2
FeSSIF solubility (µg/mL)	28.4

### Stability and degradation pathways of VTX

Understanding of a compound’s stability under various stress conditions is critical to fabricate an optimal formulation that preserves efficacy throughout its shelf life. Short-term and long-term stability studies can establish appropriate storage and handling practices to minimize compound degradation and maintain formulation quality. Zigart et al. conducted forced degradation studies of VTX following international conference on harmonization guidelines, exposing the compound to acidic, basic, oxidative, photolytic and thermolytic conditions over 14 days [22]. VTX exhibited 40% degradation after 14 days of exposure to 1 M hydrochloric acid, compared to 60% degradation under 1 M sodium hydroxide over the same period [22]. This evidences that VTX exhibits lower stability under basic conditions compared to acidic [22]. Meanwhile, exposure to H_2_O_2_ and 4,4′-azobis (4-cyanopentanoic acid (oxidative stress initiators) at 50⁰C only resulted in a degradation of 10% over 14 days, evidencing a low susceptibility of VTX to oxidation. An elevated temperature of 50⁰C as a sole stressor did not result in significant degradation [22].

### Pharmacokinetic properties of orally administered VTX

VTX has been trialled for various types of hematologic malignancies, with VTX pharmacokinetic profiles (PK) appearing independent of the specific pathophysiological type of hematologic malignancy [23]. This enables robust assessment of dosing, presence of food, steady-state exposure, and the influence of CYP3A inhibitors on PK parameter, across various indications. PK properties of a 400 mg dose of VTX under low-fat meal intake, akin to the clinical dosing regimen in humans with relapsed/refractory CLL are listed in Table 2.

**Table 2:** Steady-state VTX PK parameters under low-fat conditions for a 400 mg dose in patients with haematological malignancies [23]

Pharmacokinetic parameter	Measuremen**t**
T_max_ (h)	6
Steady-state C_max_ (μg/mL)	2.1
C_trough_ ug/mL	0.6
C_max_ (µg/mL)	2.1 ± 1.1
AUC (µg.h/mL)	32.8 ± 16.9
CL/F (L/h)	16.5
Volume of distribution (L)	256—321
Protein binding	Highly bound to human plasma proteins (> 99.9%)
Half-life (t_1/2_)	14—18
Excretion or metabolism	< 0.01% of drug excreted in urine; hepatic elimination is primary clearance route
Food effect	3.4-fold increase after low-fat meal and fivefold increase after high-fat meal, compared to fasted state

Liu et al. investigated the absorption, metabolism, and elimination of VTX to healthy female participants who received a single radioactive oral dose of VTX under fed conditions [24]. Plasma, metabolite, faecal and urine profiling revealed 100% recovery (± 5%) of the total administered dose. Mass balance analysis indicated an extent of absorption of 65%, with hepatic clearance as the dominant route of clearance and faecal excretion as the primary route of elimination. Lipophilic drugs are predisposed to metabolism by cytochrome P450 (CYP450) enzymes in the gut lumen and liver [9]. VTX is metabolised via CYP3A4-mediated mono-oxygenation to M5 involving addition of a hydroxyl group, followed by cyclization of the α‐carbon of piperazine to form M27 [25]. Other metabolites formed by CYP3A4 include M2, M3 and M4, however M27 is the major metabolite present at only 12% of total plasma drug-related material within the systemic circulation [24, 25]. However, no pharmacological activity is expected of the metabolite due to a 22-fold lower potency in inhibiting Bcl-2 proteins, thus posing no significant concerns to its efficacy or safety profile [9]. Metabolites accounted for 78.6% of the administered dose, whereas 20.8% was recovered as the parent drug [24].

Exposure of VTX at steady-state has been shown to increase proportionally over the dose range of 300–900 mg. Non-linear kinetics was observed at higher doses due to the profound dissolution-limited solubility of the drug (slow dissolution kinetics limit the apparent solubility) [23]. Transporter interaction and drug-drug interaction studies revealed the role of metabolising enzymes and P-gp and BCRP efflux transporters on the in vivo fate of VTX [26–29]. These interactions are predicted to have implications for absorption only and no systemic effects due to high protein binding [9].

The commercial formulation, Venclexta^®^, is available as 10, 50 and 100 mg film-coated tablets and with clinical doses approved up to 800 mg/day, eight tablets must be consumed. More details on the excipients and the role within the formulation are elucidated in Sect. "Amorphous solid dispersions". The 100 mg tablet has large tablet dimensions (17.2 mm long, 9.5 mm wide, oblong biconvex shape) as opposed to lower-strength 10 mg (6 mm diameter, round biconvex shape) and 50 mg (14 mm long, 8 mm wide, oblong biconvex shape) [30]. To improve patient experience with the current dosing regimen, three Phase I bioavailability studies were conducted in healthy females to assess the interchangeability of lower-strength tablets and oral suspension powder formulations in comparison to the commonly used 100 mg tablet [30]. The use of lower tablet strengths and oral powder formulations met the bioequivalence criteria (0.9–1.25) to the 100 mg tablet with respect to the AUC_t_ [30]. Film-coating of tablets can enhance stability, shelf-life of the compound, and can mask unpleasant taste and appearance for improving patient adherence. Salem et al. confirmed film-coating of Venclexta^®^ did not influence VTX exposure by conducting a study in healthy patients comparing film-coated and uncoated tablets, which revealed that both tablets were bioequivalent [31].

### Pharmaceutical food effect on VTX absorption

**Fig. 2:**
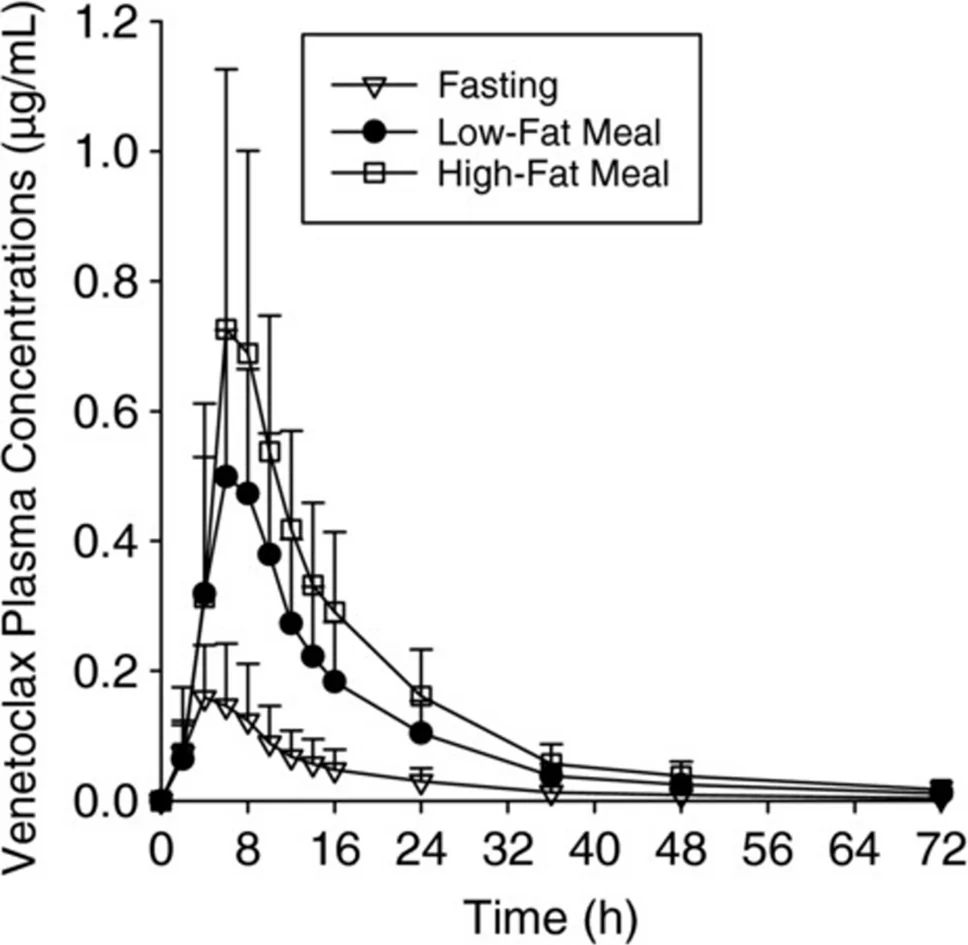
Plasma concentration–time profiles of VTX following a single 100 mg dose in fasting, low-fat meal and high-fat meal conditions in healthy subjects. Mean ± SD, n = 24 [31] (Figure reused with permission from author/s)

Evaluating the intake of food alongside lipophilic, anti-cancer therapeutics is necessary to ensure accurate dosing instructions for patients and to avoid therapeutic inefficacy and toxicity. A food-drug interaction study was conducted in heathy patients to assess the food-effect of Venclexta^®^ under fasting, low-fat, and high-fat meal conditions (Fig. 2). VTX exposure increased by approximately 3.4- and 5.2-fold following intake of a low-fat and high-fat diet, respectively, compared with fasting conditions. A delayed T_max_ was also observed under fed conditions and was attributed to delayed gastric emptying following food intake [23]. However, as difference in exposure between low-fat and high-fat compared to fasting conditions was non-significant, specifying the required fat content in a meal for maximised bioavailability is not required. The maximised bioavailability of VTX through the intake of food was taken advantage clinically by instructing patient to administer VTX with food, as outlined in the prescribing information [32].

Co-administration of VTX with food leading to enhanced exposure can be categorized into two mechanisms. Dynamic food-induced changes in gastrointestinal physiology can alter the composition and volumes of luminal fluids, inhibit CYP enzymes and efflux transporters, delay gastric emptying, accelerate transit in the small intestine, and introduce endogenous and exogenous solubilizing components [33, 34]. Such factors ultimately influence dissolution thereby affecting the rate and extent of oral drug absorption [33]. In vitro studies of VTX solubility in FaSSIF and FeSSIF media by Choo et al. exhibited similar solubility in both types of media and no evidence of a food effect [35]. This observation led the researchers to consider intestinal lymphatic transport as a possible alternative drug transportation pathway occurring for VTX in the presence of food, and investigated this mechanism using the conscious thoracic lymph-cannulated dog model (TDC) [35]. Intestinal lymphatic transport deviates from the standard portal vein pathway, and is observed for compounds with a logP value above 4.7 and a long-chain triglyceride solubility above 50 mg/mL [36]. The observation that concluded lymphatic transport was influencing the systemic exposure of VTX was a twofold decrease in plasma concentrations of conscious thoracic lymph-cannulated dogs compared with intact dogs, accompanied by a gradual increase in lymph exposure over eight hours [35]. Meanwhile, this correlated with tenfold decrease in lymphatic transport in fasted conscious thoracic lymph-cannulated dogs [35]. The maximised bioavailability in fed conditions clinically and preclinically was attributed to lipids in food that form abundant triglyceride-rich lipoprotein within enterocytes, that may be able to access lymphatic transport pathways [35].

## Oral formulation strategies applied to VTX

**Fig. 3:**
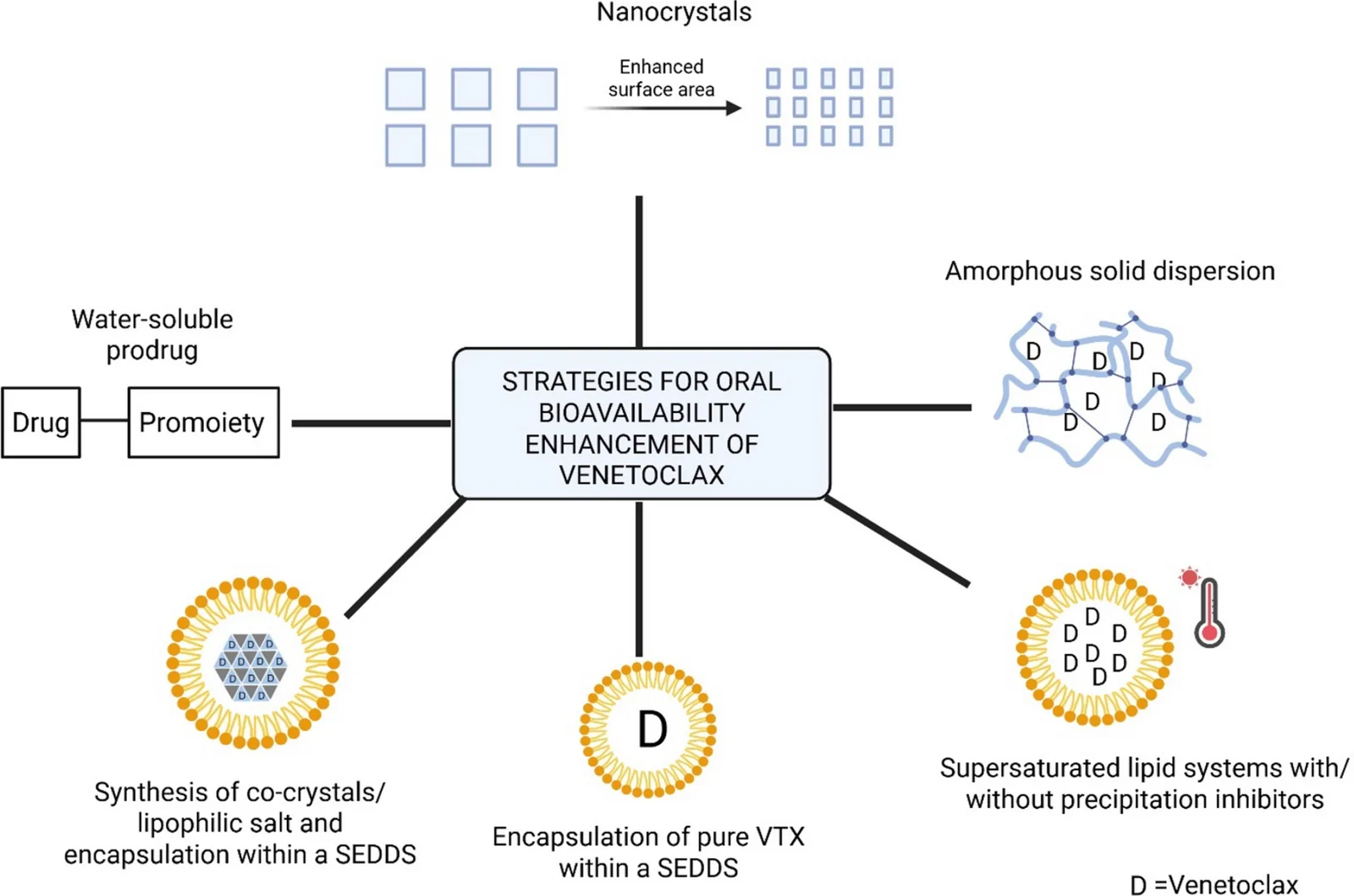
Current in vitro and in vivo trialled and published oral formulation strategies of VTX (Created with biorender)

The disposition study conducted by Liu et al. evidenced an extent of absorption of 65% VTX following oral administration, however this was conducted under fed-conditions, which is known to increase systemic exposure by 3- to fivefold. Fasting conditions will significantly reduce the amount of drug absorbed, thereby limited oral bioavailability can be said to be primarily driven by challenges in absorption, as opposed to clearance mechanisms [35]. Thereby, formulation scientists can focus their solutions on improving absorption-related factors such as solubility-limited dissolution and intestinal permeability. The goal of exploring alternative VTX formulation to the current marketed product is to overcome current disadvantages in terms of delivery, optimised release and dissolution performance, and achieve either bioequivalence or enhanced bioavailability to enable overall dose reduction. Use of innovative formulation strategies may overcome the current clinical challenges faced by patients. Herein, such novel approaches are discussed and critically appraised, focusing on the applied formulation strategy in context of formulation design the revealed impact and/or benefit (Fig. 3).

### Amorphous solid dispersions

Amorphous solid dispersions (ASDs) are a notable solubility-enhancing technology and consist of the active pharmaceutical excipient (API) dispersed in an amorphous state with a stabilising polymer excipient [37, 38]. Reducing a coarse dispersion of API to a colloidal or molecular dispersion benefits drug dissolution and absorption [38] [39]. ASDs can alleviate the consequences of oral administration of poorly water-soluble drugs as elucidated by Gala et al. with three kinase inhibitors (vemuafenib, regorafenib, everolimus), one polymerase inhibitor (Olaparib) and the commercial product of VTX (marketed as Venclexta^®^) currently approved as ASDs [37]. A patent filed by Rao et al. (Laurus Labs Limited) has been disclosed on the preparation of an amorphous form of VTX with high product yield, purity, and desirable stability [40]. Formulating a high-performance ASD requires critical consideration of various factors throughout development including drug load, polymer/surfactant selection, surfactant/polymer quantity, extrusion temperature, and appearance of the extrudate [41]. In efforts to formulate Venclexta^®^, copovidone was screened as the polymer with two surfactants (Tween 80 and Vitamin E TPGS) for drug loading and dispersibility [41]. Vitamin E TPGS resulted in a dispersion of VTX at 10% drug loading whereas the same quantity of Tween 80 enabled a dispersion of VTX at a higher drug loading of 12% [41]. Hence, the leading formulation was produced through a melt-extrusion method at 150 °C and contains the following excipients: copovidone (stabilising polymer), colloidal anhydrous silica (glidant), Tween 80 (surfactant to aid release), sodium stearyl fumarate (lubricant), calcium hydrogen phosphate (binder and filler), iron oxide yellow (coating pigment), polyvinyl alcohol (film coating excipient), macrogol 3350 -purified talc (film coating excipient), and titanium dioxide (film coating excipient) [31]. Venclexta^®^ is reported to have an oral bioavailability of 5.4% in the fasted state, which increases to 18%—28% under fed conditions due to the observed food-effect in clinical practice [10] [42].

Despite commercial approval of ASD of VTX, Bohsen et al. has continued investigations in this space to understand the influence of formulation excipients on the dispersive behaviour of ASDs [43]. Bohsen et al. evaluated three ASDs with varying concentration of VTX and characterized their precipitation and micelle formation behaviour. All three formulations exhibited acceptable dispersion behaviour, however analysis of particle size of the three dispersions exhibited significant differences [43]. ASDs prepared via melt extrusion containing copovidone as the polymer exhibited higher precipitation of VTX and less association with micelles, whilst ASDs prepared via spray drying containing hydroxypropyl methylcellulose acetate succinate as the polymer exhibited greater micelle formation and predominant association of VTX with the micelles [43]. These observations suggest that tuning the composition of an ASD can further optimise the delivery and performance of VTX.

### Nanocrystals

Forming drug nanocrystals is a well-documented solubility-enhancing approach in literature. In recent years, researchers have proven its expanded application for active targeting, delivery through different administration routes, and formulation into various atypical dosage forms [44]. Nanocrystals are nanoscopic crystals of drug compounds that do not consist of a carrier i.e. carrier free delivery systems however require surfactants for appropriate stabilisation [44]. Currently, no anti-cancer drug has been marketed as a nanocrystal, however, eleven non-oncology therapeutics across various indications have been approved [45]. A top-down manufacturing approach is commonly used by the pharmaceutical industry for marketed drugs which involves a milling or a high-pressure homogenisation process. In this process, selection of a stabiliser with appropriate viscosity alongside optimisation of process conditions (applied pressure, homogenization cycles, temperature) are critical to obtain nanocrystals with desired characteristics. Particle size reduction/optimisation is a key factor that influences oral drug absorption and systemic exposure [46]. Kala et al. obtained stable and high-performing nanocrystals of VTX using high-pressure homogenization and screening aforementioned factors based on particle size [42]. Polyvinyl alcohol (PVA) was selected as the stabilizer due to no observed alteration of melting point of VTX, suitable compatibility with the drug, and formulations with minimal viscosity for homogenization were produced. Optimum variables that led to a desired particle size of 350 nm were 2000 bar pressure and 15 cycles for homogenization, concentration of 3% of PVA as the stabilizer, and 4% trehalose as the lyoprotectant [42]. The VTX nanocrystals showed a 20, 2.3 and twofold enhancement in saturation solubility, dissolution rate and oral bioavailability in rats, respectively, compared with free drug [42]. Enlarged surface area and high dissolution rate are well-known characteristics of nanocrystals, additionally, they exhibit increased adhesiveness to intestinal cell membranes which enables a higher concentration gradient and contact time in the GIT, all factors translating to improved absorption [47]. The final lyophilized formulation by Kala et al. also exhibited high stability in gastrointestinal fluids with no change in particle size. Accelerated stability studies indicated no changes in polydispersity index, drug content or physical appearance [42].

### Lipid-based drug delivery systems

Lipid-based drug delivery systems (LBDDS) refer to a variety of lipid-containing systems with different compositions and self-assembling structures. A vast array of literature showcases the use of lipid-based formulations (LBFs) as a viable strategy for drugs that sit beyond Lipinski’s “rule-of-five” space [48, 49]. Various case studies of traditional chemotherapeutics (paclitaxel, docetaxel, doxorubicin, abiraterone acetate, SN-38) using LBDDS have demonstrated enhanced oral delivery and performance [50–53]. Readers are referred to a detailed analysis by Feeney et al. of the history of LBDDS over the past 50 years, current trending technologies, in vitro characterization tools, ending of with a perspective on the near future of these systems [54]. The success of lipid-based systems is demonstrated by superior pharmacokinetics, efficacy, and safety compared to intravenous paclitaxel, in an in vivo tumour and clinical study [55]. For this reason, translation of lipid-based systems to VTX deserves explorations and holds tremendous potential for advancement. However, only a limited number of studies have investigated LBDDS so far for VTX. Currently published studies utilising LBDDS will be discussed in the following sections, with a focus on the influence of design parameters on in vitro activity and in vivo pharmacokinetics of VTX.

#### Self-emulsifying drug delivery systems

Self-emulsifying drug delivery systems (SEDDS) are isotropic mixtures of oils, solid or liquid surfactants, or alternatively one or more hydrophilic surfactants and co-solvents, that spontaneously form oil in water (o/w) micelles under gentile agitation in the GIT [56]. A lipid-classification system was established by Pouton to differentiate between SEDDS with varying proportions of lipids and surfactants [56]. Type I formulations consist of purely oil and require digestion whereas SEDDS are representative of a Type II formulation, comprising of lipids and hydrophobic surfactants. However, with increasing hydrophilicity and quantity of surfactants, self-microemulsifying drug delivery systems (SMEDDS) and self-nanoemulsifying drug delivery systems (SNEDSS) are formed (Type III formulations) [56]. Type IV formulations contain no lipid and predominately hydrophilic surfactants and cosolvents [56]. Currently, 21 drugs utilising lipid-based systems are approved commercially and from these, 16 drugs utilise Type I LBDDS, 3 drugs utilise Type II, 5 drugs utilise Type III and 2 drugs as Type IV [57]. From this, five are oncology therapeutics namely tretinoin (Vesanaoid), bexarotene (Targretin), enzalutamide (Xtandi), nintedanib (Ofev) and midostatrin (Rydapt).

A dataset was compiled of 668 unique SEDDS formulations of 20 poorly soluble drugs showcasing their heavy utilisation in drug delivery [58]. However, only one notable SEDDS formulation of VTX by Rajana et al. is reported in the literature underlying the potential of unexplored compositions and formulation-enhancing features (as discussed in Sect. "Expansion of the lipid-based formulation platform") for further improvement [59]. Rajana et al. utilised design of experiments (DoE) to form an optimum SEDDS of VTX by studying the influence of quantity of oil, surfactant, and co-surfactant, stirring rate, and stirring time on globule size, polydispersity index (PDI), self-emulsification time, and % transmittance of formed emulsions [59]. DoE is a commonly used approach for evaluating the influence of formulation-related parameters for desirable dispersion, dissolution kinetics and overall biopharmaceutical performance. To select appropriate lipids and surfactants, solubility and emulsification studies were performed [59]. The final formulation consisted of cinnamon oil, Cremophor RH 40 and Transcutol P in a ratio of 100: 300:300 and exhibited a globule size of 71.3 nm (after reconstitution), PDI of 0.112, emulsification time of 16 s, a transmittance of 92.55% and an encapsulation efficiency (EE) of 94%. In the SEDDS preconcentrate, a higher surfactant fraction compared to lipid enabled a small globule size primarily increasing interfacial surface area for dispersion in GI fluid, solubilisation and permeation during GIT transit [26]. This was proven during their in vitro drug release study, whereby release of VTX from SEDDS displayed a 7.3-fold enhancement compared to free drug, fitting into zero-order release kinetics. High stability was also observed of the formulation with non-significant minor differences to globule size, PDI and encapsulation efficiency over 60 days. LBFs, particularly SEDDS, are known for forming colloidal species that enable solubilisation and supersaturation drug effects for enhanced absorption [60]. Increased drug solubilisation and generated transient supersaturation during digestion can counteract the varying pH conditions of the GIT. Rajana et al. reported that under varying pH conditions (pH 1.2, 4.6, 6.8 & 7.4), use of VTX-SEDDS maintained drug solubilisation from 60–80% with no significant drug precipitation at any pH value [59]. Due to encapsulation of drug in the lipid based colloidal structures as opposed to free drug enabled VTX to remain solubilised in the different pH conditions. This translated to enhanced oral bioavailability, as demonstrated by C_max_ and AUC_0–24_ PK parameters being enhanced by fivefold and 4.76-fold, respectively, compared to VTX suspension [59].

Rajana et al. were also the first to report on evaluation of in vitro anti-cancer efficacy of VTX-loaded SEDDS in breast cancer cells [59]. The selection of this cell line can arise as in addition to leukemic cancer cells, Bcl-2 proteins are also involved in the survival and proliferation of solid tumours including breast cancers and small-cell lung cancer (SCLC). In MDA-MB-231 (breast cancer cells), the IC_50_ value for free VTX and VTX-SEDDS was 9.9 µM and 3.3 µM, respectively, observing a threefold enhanced cytotoxicity when using the SEDDS formulation. Qualitative assessments of formulations using fluorescence microscopy for cell uptake, nuclear morphology and production of reactive oxygen species generation were also performed. VTX-SEDDS exhibited greater fluorescence compared to raw drug at their respective IC_50_ values in all three cases, proving the high cytotoxic effects and damage expedited by the formulation compared to free drug. Explicit alteration in morphology of cells was observed in treated cells including disruption of cell membrane, shrinking of the cell and notable reduction in live cells, as compared to the control group [59]. With Bcl-2 as an anti-apoptotic protein, confirmation of the potency of VTX-SEDDS via protein expression was studied. Their findings were consistent with published mechanisms of VTX-induced apoptosis through a BAX-dependent pathway in vitro [59].

#### Supersaturated LBFs and precipitation inhibitors

Supersaturated lipid-based formulations (sLBFs) contain drug concentrations above their equilibrium thermodynamic solubility [61]. This approach involves a heating–cooling method whereby the formulation is thermally heated up to a certain temperature (50 °C – 60 °C) for 20 min – 24 h to saturate the drug above its equilibrium solubility and subsequently cooled down to ambient temperature [20, 61–63]. Koehl et al. employed a heating method for supersaturation of Type I (oil-based i.e. olive oil, Peceol, Captex 100 and Capmul MCM) LBFs of VTX [20]. All oils displayed enhancement in VTX concentration after heating compared to solubility at 37 ⁰C, however Peceol-based sLBF displayed the greatest enhancement and maintained a supersaturated concentration for 20 days [20]. During in vitro dispersion, the Peceol-based sLBF displayed the highest recovered amount of VTX in the aqueous phase compared to a pure drug aqueous and Peceol suspension [20]. In addition, recovered VTX was significantly higher in the lipid phase compared to the solid and aqueous phase. The lipid phase consisting of undigested or partially digested lipids also contained absorbable VTX particularly as the drug can partition into the aqueous phase, be absorbed via the lipid absorption pathway, and released through further digestion of Peceol, in vivo. This translated to a 4.29-fold improvement in bioavailability compared to free drug powder during an in vivo pharmacokinetic study in pigs. To enhance the stability of sLBFs by reducing the risk of drug precipitation upon digestion, Koehl et al. further investigated a variety of precipitation inhibitors to prolong supersaturation [64]. In silico tools such as drug-excipient mixing enthalpy and in vitro PI solvent shift test/s were incorporated for screening compatible precipitation inhibitors [64]. However, the study displayed showed a lack of *in-vitro-in-vivo* correlation, although solubilisation studies displayed prolonged supersaturation by inclusion of precipitation inhibitors, this did not correspond to improvement in in vivo bioavailability [64]. This was the first in vivo study that tested precipitation inhibitors inclusion in a Type I supersaturated LBF, whereby previous studies explored “supersaturable” Type III and Type IV formulations. Type III/IV formulations have a greater risk of precipitation upon dispersion due to the hydrophilic excipients, compared to Type I LBF that contain only an oil excipient. Inclusion of a precipitation inhibitor was deduced to be not required for a Type I sLBF due to low precipitation risks and sufficient supersaturation duration for enhanced absorption [20]. Inclusion of a precipitation inhibitor enhanced formulation viscosity and solubility in the sLBF, leading to increased trapping of drug and reduced partitioning/diffusion into the aqueous phase [20]. Moreover, the *in-silico* software utilised for screening did not account for the complex interactions of the precipitation inhibitors-drug complex with formulation excipients and GI fluids. To revive the benefits of precipitation inhibitors, shifting to a composition with reduced lipid and more hydrophilic excipients would be required [20]. Utilizing a combined dispersion/absorption set-up can further benefit formulation performance and provide a more accurate IVIVC [20].

#### Lipophilic salts in SEDDS

Poorly water-soluble compounds can be divided into two categories i.e. “grease ball” compounds that have a high logP and exhibit high lipid solubility and “brick dust compounds” which have high crystallinity, require a large clinical dose and do not dissolve easily in lipid. Application of SEDDS for “brick-dust” compounds remains largely limited, highlighting the pressing need to enhance the solubilizing capacity of excipients to accommodate the required drug dose [11]. Lowering a drug’s high intramolecular properties by pairing it with lipophilic counterions can enable enhanced solubility in LBFs and aqueous media [11]. Production of a lipophilic salt requires appropriate counterion selection and is based on its ability to disrupt the crystal packing of the drug. Ideal physiochemical features of counterions include high molecular weight, steric bulk, minimal hydrogen bonding potential, an asymmetrical structure and presence of minimal heteroatoms [11]. For detailed insights on the design, synthesis, characterization and broad advantages of lipophilic salts, readers are referred to a comprehensive review by Willams et al. [11].

A largely ignored feature of lipophilic salts is toxicity which makes it crucial to select counterions that are FDA approved in the “Generally Regarded as Safe” (GRAS) list. Docusate as a GRAS excipient is one of the most widely utilised counterions. However, due to its physiological effect as a laxative, Ryan et al. assessed the utility of alternative counterions alongside docusate for the formation of lipophilic salts of VTX, their loading into SEDDS and their biorelevant in vitro digestion performance [65]. The resulting library of lipophilic salts and the appropriate characterization performed in terms of their melting point, biorelevant solubility, equilibrium solubility in SEDDS is summarised in Table 3.

**Table 3:** Physical characteristics and formulation properties/parameters of synthesised lipophilic salts of VTX adapted from [65]

Lipophilic salt/s	Melting point (⁰C)	Purity (%)	Biorelevant solubility (mg/mL)	Equilibrium solubility in medium-chain SEDDS (mg/g)
VTX	140	100	5.5	5.5
VTX docusate	151	98.76	16.2	48
VTX octadecyl sulcate	88	99.14	8.4	39
VTX dodecyl sulfate	91	98.91	10.9	45
VTX decyl sulfate	101	99.55	10.3	36
VTX octyl sulfate	109	97.31	7.7	39
VTX octane sulfonate	90	99.02	7.4	28
VTX dodecane sulfonate	112	97.02	9.8	45

High purity values confirmed the successful formation of lipophilic salt/s with the correct 1:1 stoichiometric ratio between pure VTX and its respective counterions [65]. All lipophilic salts showed comparable enhancement in solubility in biorelevant media and in a medium-chain SEDDS compared to pure drug, proving the suitability of the counterions for lipophilic salt synthesis [65]. Lipophilic salts are known to have decreased crystal energy which can be reflected by a lower melting point [65]. Melting point of the synthesised lipophilic salts and solubility in the SEDDS did not display a direct correlation largely due to the notion by Koehl et al. that the solubility of lipophilic salts in SEDDS can also be determined by their solvation characteristics and lipophilicity instead of purely solid-state properties [65]. This is evident in Table 3 where an observed increase in melting point of VTX-docusate did not negatively impact its solubility in biorelevant media and lipids.

In vitro dispersion and digestion data (not displayed) by Ryan et al. indicate a dispersion solubilising capacity of SEDDS of up to 0.8 mg/mL when a lipophilic salt was incorporated, exceeding the dispersion solubility of VTX free base at 0.05 mg/mL. All counterions displayed comparable solubility and in vitro performance. However, only the *in-vivo* pharmacokinetics of VTX docusate in a SEDDS was investigated by Koehl et al. in pigs [66]. The formulation displayed an in vivo bioavailability in landrace pigs of 30.8% ± 12%, marking a significant threefold increase in bioavailability compared to the commercial formulation in the fasted state [66]. Replacing a long chain oil with a medium chain oil (Capmul MCM) reduced the bioavailability of VTX to 25.1 ± 5.7% whilst the use of a surfactant-only formulation further lowered the value to 20.2 ± 4.6%. All three lipophilic-salt SEDDS displayed a similar area under the curve (AUC) and non-significant difference in bioavailability to Venclexta^®^ in the fed state, signifying that all SEDDS were able to mimic the food effect [66]. Lipophilic salt synthesis approach has gained attention for various other “brick-dust” (mebendazole, nilotinib and rebamidpide) and non-brick-dust compounds (erlotinib, gefitinib, cabozantibin, atazanavir, itraconazole, metformin) ([67–69]. The approach effectively transformed them to counter ion complexes to disrupt the high crystalline properties of the compounds enabled and enhanced solubilisation and pharmacokinetic profiles compared to pure base lipid suspension [67–69]. Thus, encapsulation of a lipophilic salt within a SEDDS offers multiple advantages, combining high in vitro solubilisation with superior in vivo pharmacokinetic performance.

### VTX prodrug

**Fig. 4:**
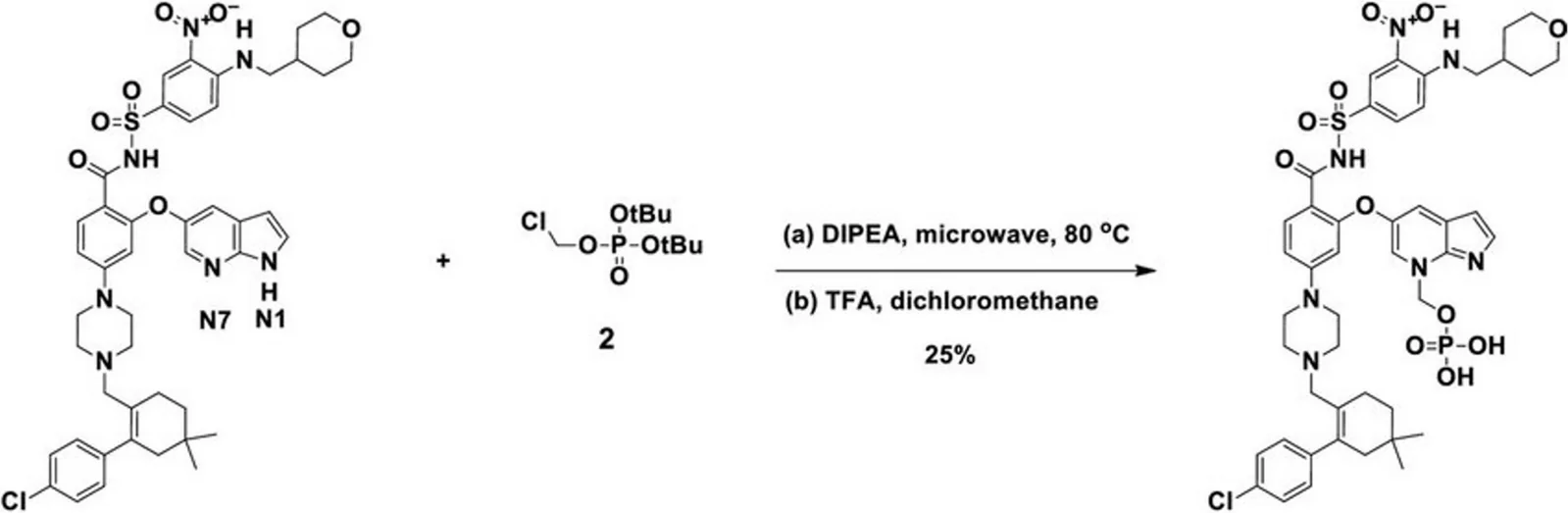
Synthesis pathway for the conversion of VTX to prodrug, ABBV-1667 (Figure reused with permission from authors)

Prodrugs are inactive derivatives of a parent drugs that are converted to their pharmacologically active agent upon in vivo enzymatic or physico-chemical transformation. Saleem et al. added a liable and ionisable phosphate pro-moiety to generate a novel prodrug of VTX, ABBV-1667 (Fig. 4), and thus efficient transformation to the active drug was enabled in vivo by alkaline phosphatases [70]. Inclusion of a phosphate functional group enhanced water solubility 1000-fold (3.5 mg/mL compared to 0.0004 mg/mL), this favourable property enabled generation of a high drug-load immediate release tablet with lower size dimensions compared to the commercial tablet for an equivalent dose [70]. Following intravenous administration, ABBV-167 rapidly and extensively converted to VTX in both mice and dogs with less than 1% of ABBV-167 remaining in systemic circulation [70]. Oral administration of ABBV-167 enabled higher VTX C_max_ and AUC_∞_ values by 2.1 and 1.9-fold in the fasted state, compared to pure venetoclax tablets [70]. Moreover, a food effect was also observed which enabled higher VTX C_max_ and AUC_∞_ values by 1.7 and 2.1-fold in the fed state compared to fasted. This observed food-effect was lower compared to the reported food effect of the commercial tablets, however, needs to be confirmed with a greater population size for statistical confirmation [70]. To date, ABBV-1667 is the only reported prodrug of VTX.

## Alternative, advanced formulation strategies for Venetoclax

Advancing currently trialled formulations by exploring alternative compositions or combining multiple bio-enabling strategies, as well as investigating inorganic-based vehicles, may unlock additional enhanced biopharmaceutical performance of VTX. Improvement in terms of formulation solubility and stability, drug release kinetics, and drug permeability can result in improved pharmacokinetics and thus improved pharmacodynamic parameters clinically. Given that four LBFs have been reported for VTX, indicates that further exploration of advanced formulation approaches is warranted particularly considering the recent breakthrough discovery of the in vivo efficacy of VTX for HIV and estimated upcoming broader adoption/s that are under investigation. SEDDS offer the most potential for repurposing for HIV indications, considering they have been shown to mitigate the food-effect [66]. Higher absorption into plasma and target organs (depending on the indication) and lower exposure to non-target organs can enhance the efficacy-toxicity balance and translate to progress the delivery and performance of a Bcl-2 inhibitor. Notably, various SEDDS systems of other drugs have been assessed in vivo for their biodistribution properties*,* demonstrating minimal accumulation in non-target organs and higher accumulation in target organs [71–73]. However, previously studies of VTX have not evaluated biodistribution in various tissues compared to the commercial product or the free drug, evidencing a clear future direction into how novel delivery systems of VTX impact tissue distribution.

### Untrialled formulation approaches, ionic liquids and further characterization

In this section, formulation strategies and/or formulation characterization conducted with other poorly water-soluble and/or brick-dust molecules for oral delivery within the literature will be discussed, with a reasoning of applying such techniques to VTX i.e. i) stability and advanced characterization of lipophilic salts ii) mesoporous silica nanoparticles (MSNs) iii) polymeric nanoparticles iv) solid lipid nanoparticles (SLN) and nanostructured lipid carriers (NLC) v) solidification of LBFs vi) supersaturation of LBFs vii) silica-lipid-hybrid (SLH) microparticles and viiii) super-SLH. This section will conclude with a summary of in silico tools used to facilitate formulation development and enable clinical translation of LBFs.

#### Stability and cytotoxic characterization of lipophilic salt/s and ionic liquids

As mentioned in Sect. "Lipophilic salts in SEDDS", lipophilic salts of VTX have been synthesised, characterized and investigated for in vitro solubility and in vivo pharmacokinetics in pigs. A key future direction involves formally assessing the long-term stability of lipophilic salt-based LBFs during storage. Lai et al. reported that the use of weakly acidic counterions can lead to salt disproportionation within the formulation and precipitation of the formulated compound. Addition of strong acids to formulation, including heptafluorobutyric acid and dichloroacetic acid, enhanced the ionisation of the compound that led to highly stable lipid soluble complexes being formed [74]. Advantages of lipophilic salts can extend beyond the benefits of drug absorption as shown in studies by Moshkiur et al. and Saeed et al. [75, 76]. Ionic liquids, a related form of lipophilic salts, are considered more favourable due to greater disruption of the crystal lattice and often display a melting point of less than 100 °C [66]. A comprehensive study by Moshikur et al. synthesised ionic liquids of methotrexate (MTX) and evaluated their anti-cancer activity in a HeLa mammalian cancer cell line compared to MTX sodium salt. A tetrabuyulphosphonium-based and tetramethylammonium -based IL of MTX displayed significantly low IC_50_ values as presence of a long alkyl chain and benzene ring, respectively, enhanced interactions with the cell membrane and led to greater penetration. Meanwhile, cholinium-based IL of MTX displayed relatively low cytotoxicity due to more hydrophilic hydroxyl groups. Oral administration of IL-MTX revealed enhanced exposure and a lower accumulation in the intestine, lungs and heart compared to MTX sodium salt. Moreover, IL-MTX inhibited tumour growth significantly compared to the PBS and MTX sodium treatment groups. Saeed et al. also synthesised lipophilic salts of metformin to enhance its repurposing for cancer therapy, whereby metformin docusate exhibited 700- to 4300-fold improved potency compared to metformin hydrochloride [77]. The relevance of testing lipophilic salts/ionic liquids on cell lines can be debated in context of oral delivery, considering these salts typically dissociate prior to absorption.

#### Mesoporous silica nanoparticles

Inorganic mesoporous silica-based nanoparticles (MSNs) have gained interest as matrixes for delivery of poorly water-soluble drugs (PWSDs) due to their biocompatible nature, variable surface functionalization properties, tuneable structure and composition and cellular targetability [78, 79]. Confinement of the drug within interior pores in an amorphous or molecular form as opposed to crystalline facilitates enhanced absorption and bioavailability [80] The high hydrophilic surface area of MSNs also aids wetting and dispersion of the encapsulated drug [80]. Use of MSNs for PWSDs has resulted in enhanced in vitro dissolution translating to enhanced in vivo performance of many chemotherapeutics [78, 81]. MSNs with small pore size and unmodified surfaces can present challenges for VTX’s large size and brick-dust properties leading to low loading, poor pore entry, risk of drug crystallisation and poor release properties. Use of silica materials with larger pores and surface-functionalisation can improve loading, reduce crystallisation within pores and enhance release [82, 83]. An example can specifically be observed with furosemide, a BCS Class IV compound that resulted in improved release profiles compared to crystalline drug and commercial formulation when encapsulated within SBA-15 mesoporous silica. SBA-15 is constituted by hexagonally ordered tuneable uniform mesopores (4 −14 nm) and thick silica pore walls for high hydrothermal stability [84]. Utilisation of SBA-15 for furosemide achieved 71% drug release at 2 h dissolution compared to 49% from the commercial product [84]. Surface functionalisation in the form of phosphate groups can further enhance loading by improving electrostatic drug-carrier interactions, resulting in improved solubility of a BCS Class IV drug i.e. vorinostat (a BCS Class IV drug) by 2.6–4.3-fold compared to non-functionalized particles [85]. Paclitaxel, a widely studied BCS Class IV compound, has been formulated in various types of MSNs including surface-functionalized for passive and active cancer targeting and as a combination therapy for overcoming multidrug resistance [81].

#### Polymeric nanoparticles

Advances in polymer chemistry have transformed the field of polymeric nanoparticles for use in drug delivery applications [86]. Various types of polymers such as natural, synthetic, biodegradable and bioabsorbable polymers have been utilised for efficient delivery of PWSDs [86]. A polymeric mixed micelle based on Pluronic F-127 and alpha tocopherol polyethylene glycol 1000 succinate (TPGS) as excipients was formulated with VTX [87]. This remains the only reported polymeric formulation of VTX and despite the enhanced cytotoxicity and release, it was designed for intravenous (IV) delivery as opposed to oral delivery [87]. Key challenges when loading “brick-dust” compounds in polymeric nanoparticles include the tendency for nucleation and crystal growth, as well as appropriate dispersibility, release and absorption. Approximate excipient selection for successful polymeric nanoparticle formation was applied in studies involving hydrochlorothiazide and paclitaxel. Hydrochlorothiazide is a BCS Class IV compound that was developed into a polymeric nanocoacervate using chitosan. Critical polymer selection was observed with chitosan as its available free functional groups enable cross-linking interactions with the drug [88]. The optimised system displayed a small particle size with narrow polydispersity and revealed a linear diffusion profile of hydrochlorothiazide during release studies [88]. Similarly, a self-assembling polymer, namely N-palmitoyl-N-monomethyl-N,N-dimethyl-N,N,N-trimethyl-6-O-glycolchitosan (GCPQ), was utilised for the oral delivery of paclitaxel. Enhanced oral uptake was attributed to mechanisms including enhanced aqueous dissolution by formation of drug nanoparticles, mucoadhesion of drug nanoparticles and uptake of drug nanoparticles bypassing P-gp efflux pumps [89]. Other strategies for optimal performance include surface modifications, optimal polymer/drug ratios and solvent selection for enhanced nanoparticle stability, mucosal adhesion and enhanced drug loading capacity. To achieve success in oral delivery of PWSDs using polymeric nanoparticles, it is critical consider how polymeric nanoparticles can overcome the barriers of oral delivery and to conduct in vivo evaluations to understand their pharmacokinetic properties.

### Expansion of the lipid-based formulation platform

#### Solid lipid nanoparticles (SLN) and nanostructured lipid carriers (NLCs)

Solid lipid nanoparticles (SLNs) and nanostructured lipid carriers (NLCs) are widely employed colloidal lipid-based carrier systems, particularly for oral delivery of PWSDs including anti-cancer agents [39, 90, 91]. SLNs are composed of one or more solid lipids to solubilise the drug and are stabilised by surfactants, however commonly displays low drug loading and expulsion of encapsulated drug during storage [90, 91]. NLCs incorporate a mixture of solid and liquid lipids to form a matrix that can accommodate higher drug loads and improve stability [90]. Nasirizadeh et al. and Galeano et al. published detailed overviews into the different production techniques of SLN and NLCS, various in vitro and in vivo (pharmacokinetics and distribution) evaluations conducted, and the role of particle structure on drug release [92, 93]. SLN and NLCs have widely been used to improve the oral bioavailability of poorly water-soluble drugs by improving dispersion and thus, absorption in the GIT [91].

Talegaonkar et al. specifically reviewed the use of SLN and NLCs for BCS Class IV drugs including cyclosporine A, amphotericin B, curcumin, decitabine, tacrolimus, etoposide, saquinavir, lopinavir and docetaxel [94]. All drugs encapsulated in SLNs/NLCs demonstrated superior pharmacokinetic performance compared to either drug suspension, drug solution or commercial product as relevant controls [94]. Across the investigated drugs, advantageous outputs also included superior performance over polymeric nanoparticles, reduced nephrotoxicity compared to the commercial product, higher in situ permeability compared to drug solution, and enhanced cytotoxicity against cancer cells in vitro [94].

#### Solid self-emulsifying drug delivery systems

SEDDS possess an innate ability for the delivery of poorly-soluble drugs, however their therapeutic potential has been limited by challenges such as inadequate stability and portability, risk of re-crystallisation of the active ingredient, low drug loading, potential GIT irritation due to their high surfactant concentration and high production costs particularly due to packaging requirements [95, 96]. To address these issues, novel solidification techniques are necessary to prolong the shelf-life stability, avoid precipitation and re-crystallisation of the active ingredient, and enable formulation of an acceptable solid dosage form [39]. Transformation of a LBF into a solid formulation can result in various biopharmaceutical advantages including improved stability, enhanced safety due to reduced surfactant concentration, improved solubilisation and dissolution, controlled drug release and industrial/commercial benefits [35]. However, the choice of solidification technique and type of solid carrier must be selected appropriately to retain high solubilisation properties. Achieving adequate re-dispersibility of solid formulations to ensure performance comparable to liquid formulations remains a long-standing challenge in formulation research [39]. Selection of solid carriers depends on their lipid adsorbing capacity, flow properties, and critically, their hydrophilic or hydrophobic nature which can play a major part in dictating the release and solubilisation potential of formulated drugs [97]. Carriers can be divided into inorganic materials (encompassing silicon dioxide, carbonates and aluminosilicates) or organic materials (encompassing polymers and nanostructured carbon) [95]. Solidification can be achieved by solidifying liquid SEDDS excipients via physical adsorption to solid carriers or stabilising dispersed emulsions via spray or freeze-drying [95]. For a detailed insight into the benefits of solidification, selection of solidification method, and the influence of drug-solid carrier and lipid-solid carrier interactions on drug dissolution and absorption, readers are encouraged to consult a detailed review by Joyce et al. [95]. Table 4 lists the various formulation parameters alongside in vitro and in vivo study outputs for a range of non brick-dust molecules. From the present studies, all solid-SEDDS generally demonstrate improved in vivo oral drug absorption compared to crystalline drug [95]. The current challenge lies in preserving their performance equivalent to their liquid counterpart [95]. Among the solid-SEDDS reporting both in vitro and in vivo characterisation, danazol and simvastatin evidenced an in vitro ranking of formulations that was predictive of their in vivo performance [98] [99]. Despite this, the literature largely lacks robust *in-vitro-in-vivo* correlations (IVIVCs) for s-SEDDS, with many studies not performing systematic comparisons in vitro and in vivo comparasions. Comparing in vitro solubilisation of solid-SEDDS with in vivo pharmacokinetics alongside advanced analysis techniques is proposed to advance the structure–activity relationship and guide the rational development of effective s-SEDDS [95].

**Table 4:** Formulation composition, solidification technique, in vitro release and in vivo pharmacokinetic study parameters of solid-SEDDS of oral delivery of poorly water-soluble drugs

Drug	Solid carrier & properties	Solidification technique	In vitro release	In vivo pharmacokinetic study
Paclitaxel [100]	Aerosil 200 (hydrophilic fumed silicon dioxide)	Spray-drying	70% dissolution in pH 6.8 buffer	1.82-fold enhancement in AUC of solid-SEDDS compared to PTX solution
Simvastatin [99]	Aerosil 200 (hydrophilic fumed silicon dioxide)	Spray-drying	Superimposable dissolution profiles of liquid and solid-SEDDS	2.1-fold enhancement in AUC of solid-SEDDS compared to marketed formulation
Cilnidipine [101]	Neusilin US2 (magnesium aluminometasilicate)	Physical adsorption	92% dissolution; significantly higher compared to pure drug and marketed formulation	In vitro absorption study from everted rat intestine displayed 98% absorption within 120 min
Furosemide [102]	Microcrystalline cellulose (synthetic polymer)	Physical adsorption	Sustained release pattern with 80% dissolution in 180 min	Gradual urine output providing sustained output and predictable therapeutic response
Docetaxel [50]	Silicon dioxide (brand specifics not listed)	Spray-drying	In vitro release profile not evaluated	sixfold higher AUC and 6.5-fold higher absolute BA compared to pure drug solution

#### Supersaturated-SEDDS (super-SEDDS)

Supersaturated systems differ significantly from super saturable systems and refer to the use of elevated temperature to enhance solubility and drug loads in SEDDS preconcentrates [103]. Nicky et al. conceptualised this strategy using a heating method involving a heating phase of 5 h at 60⁰C at concentrations above S_eq_ followed by an overnight cooling phase at 37 ⁰C, whereby complete dissolution of the drug was confirmed using polarised light microscopy. Nicky et al. formed supersaturated-SEDDS for two poorly water-soluble drugs, simvastatin and halofantrine, performed fundamental in vitro and in vivo studies that illustrated the beneficial performance of super-SEDDS [61, 104]. Critical considerations when formulating effective super-SEDDS include the heating period for elevated and maintained solubility, the extent of supersaturation that can be obtained and physical stability over long-term storage, considering a supersaturated state is unstable thermodynamically [105]. To account for these factors, formulators typically optimise and compare heating methods, study various supersaturation levels above equilibrium solubility, and investigate various stability-notifying properties after long-term storage [105]. While there are many examples of super-SEDDS and solid-SEDDS of PWSDs reported in literature, coupling advantages of both strategies within one formulation has not been widely explored to date. So far, only Moller et al. has reported the coupling of supersaturation and solid carrier-stabilised SEDDS on the in vitro solubilisation of blonanserin [106]. Increasing the supersaturation extent from 90% Eq_S_ to 300% Eq_S_ enhanced solubilisation of pure BLON, however transformation to supersaturated-solid SEDDS decreased the extent of solubilisation compared to its liquid counterpart likely due to interactions between negatively charged silica and positively charged drug [106]. Non-saturated and saturated systems, when adsorbed onto silica, can alter the biopharmaceutical performance of the drug. While solidified systems provide shelf-life stability, reduce precipitation risks and facilitate tablet formulation, the potential trade-offs in solubilisation and absorption must be carefully evaluated through in vitro and in vivo studies.

#### Silica lipid hybrid (SLH) microparticles

SLH are non-saturated solid-state systems with a single lipid-vehicle, and are fabricated by stabilising drug-encapsulated lipid nano-emulsions with porous silica nanoparticles [107]. Similarly to solidified SEDDS, SLH microparticles combine the solubilizing effect of lipids with the stabilizing property of silica nanoparticles. Lyophilization and spray-drying are commonly utilised techniques for solidifying SLH, and require careful selection of cryoprotectant, spray-drying conditions, and lipid loading level to form optimal SLH carriers [107–111]. SLH has demonstrated in vitro and in vivo efficacy for SN38, ibuprofen, celecoxib and simvastatin in the form of in vitro evaluation and pharmacokinetic studies in rodents, dogs and humans. A summary of the studies reporting enhanced in vivo drug delivery of SLH microparticles is summarised in Table 5. From the listed drugs, SN38 and indomethacin demonstrated in vitro ranking of formulation that was reflected in vivo [110, 112]. In addition, Tan et al. established IVIVCs for celecoxib by correlating dissolution efficiency at 15 and 180 min with in vivo bioavailability and C_max_, respectively [98]. The dissolution efficiency at 15 min correlated linearly with in vivo bioavailability for two SLH formulation/s with R^2^ values of 0.989 and 0.9507 [98]. Meanwhile, the dissolution efficiency at 180 min correlated linearly with the in vivo C_max_ values with R^2^ values of 0.99 and 0.96 [98].

**Table 5:** Formulation composition, solidification technique, in vitro release & in vivo/clinical pharmacokinetic study outputs for SLH systems of poorly water-soluble drugs

Drug	Excipient/s	Solid Carrier & Properties	Solidification technique	In vitro release	In vivo/clinical pharmacokinetic study
SN38 [110]	Oleylamine and Labrasol (1:9)	Fumed Aerosil 380 (hydrophilic silica)	Lyophilisation	SLH achieved 40% dissolution in 2 h compared to 10%	176% higher relative bioavailability
Simvastatin [107]	Capmul MCM and soybean lecithin	Aerosil 300 and Syloid 244	Spray-drying	In vitro release profile not evaluated	3.5-fold enhanced bioavailability compared to marketed formulation
Celecoxib [98, 111]	Capmul MCM and lecithin	Aerosil 380	Spray-drying	2- to fivefold enhancement in dissolution	6.5-fold enhanced bioavailability and desired therapeutic efficacy at a lower dose compared to an aqueous suspension
Indomethacin [112]	Lecithin or oleylamine	Fumed Aerosil 380	Spray-drying	2–fivefold enhancement in dissolution	Bioavailability was 93% compared to 53% and 64% for aqueous suspension and o/w emulsion, respectively

#### Super-SLH

SLH systems are limited to drug loads of less than 5% w/w, similar to liquid and solid-SEDDS in their unsaturated form, due to restricted solubility of the drug in the lipid phase and the silica to lipid ratio within the formulation [113]. Analogous to super-SEDDS, super-SLH involves the supersaturation of drug within the lipid by use of heat. However, they are stabilised with nano-porous silica via simple lipid absorption in contrast to the use of spray-drying for fabrication of SLH systems. Schultz et al. applied super-SLH ibuprofen as the first model compound, and a comparison between super-SLH (saturation degree of 227% S_eq_) and spray-dried SLH (%) displayed equal systemic exposure however beneficially, super-SLH enabled a 60% reduction in formulation dose due to the greater drug load [113]. Increased drug load did not enhance ibuprofen absorption, which was attributed to the presence of increased crystalline drug content, highlighting importance of the heating method to appropriately convert crystalline drug into molecularly dissolved drug [113]. In further studies, super-SLH was applied to poorly water-soluble drugs such as abiraterone acetate and fenofibrate, elucidating the impact of supersaturation, lipid type and nanostructure of solid carriers on the solubilisation and pharmacokinetics of these compounds [51, 53]. Schultz et al. formed IVIVCs of abiraterone acetate, by comparing the area under the curve (AUC) obtained from in vitro lipolysis and dissolution studies with in vivo pharmacokinetic data [53]. A weak IVIVC with a R^2^ value of 0.156 was observed between dissolution and PK data and was attributed to the lack of biorelevant dissolution media as only gastric media was utilised. The correlation largely improved when lipolysis was compared to PK data (R^2^ value of 0.53), reflecting the use of biorelevant fasted media and addition of pancreatic enzymes to enable lipid digestion [53].

## Tools to aid development and clinical translation of lipid-based formulations

### In silico screening for formulation development

Formulation development of LBFs begins with selecting suitable excipients by evaluating their solubilizing capacity for the intended drug. Experimental techniques including differential scanning calorimetry, UV–Vis spectroscopy and chromatography are commonly used to measure drug concentration in excipients, however, this method involves extensive iterative optimisation [114]. Recently, there has been growing interest in in silico tools, largely due to their predictive capacity and their ability to significantly reduce experimental workload [114]. Brinkmann et al. applied a thermodynamic modelling framework, namely the Perturbed-Chain Statistical Associating Fluid Theory (PC-SAFT), for predictive drug solubility in excipients [114]. In silico screening results agreed with experimental data, validating this method for determining most-appropriate LBFs for a given compound with low experimental effort [114]. Gao et al. showcased the utility of machine learning in the form of molecular dynamic (MD) simulations to investigate molecular interaction between excipients and drugs, and coupled with computational methods, they proved the utility of these tools for rational SEDDS design [115]. Proper integration and application of such tools can expedite early-stage development of small-molecules, enabling formulations to advance more efficiently throughout development stages [115].

### In vitro-in vivo correlations

High in vitro-in vivo correlations (IVIVC) form the basis for advancing lipid-based formulations from preclinical stages to humans [116]. Yet, a leading reason behind the limited commercial success of oral LBFs is the construction of poor or inconsistent IVIVCs [116]. This largely stems from the complex in vivo processing of LBFs that differs from conventional solid dosage forms and a lack of mechanistic understanding of LBF behaviour in vivo [116]. In some instances, formulations that displayed precipitation in vitro display non-significant difference in exposure to formulations with no precipitation, as drug can precipitate in an amorphous state in vivo and lead to redissolution [116]. Additionally, many simple in vitro models do not reflect the true physiological conditions due to the absence of an absorption component, thereby overestimating precipitation in some scenarios. Butler et al. provided a detailed overview of advanced in vitro tools used for the prediction of in vivo performance of oral dosage forms, and discussed future directions for utilising them to enhance the development of such formulations [117].

Furthermore, Meola et al. revealed that drug release from a LBF was not translated between species, as observed by the low correlation of 0.06 between rats and human exposure [107]. Poor IVIVCs alongside minimal validity of animal models are pressing issues that must be overcome to ensure clinical success. Use of advanced computational modelling such as physiologically based pharmacokinetics modelling (PBPK) has emerged as a reliable tool for supporting pharmaceutical development, establishing IVIVICs, predicting *complex *in vivo behaviour and importantly, minimising the need for excessive animal testing. PBPK modelling incorporates i) physiological, biological and genetic characteristics of the species and ii) physiochemical and disposition attributes of a dosage form mechanistically simulate and predict in vivo PK of a drug [118]. To build a PBPK model, input of organism physiology/dosing and drug/formulation parameters are required. Physiology parameters of various animal species and humans are built into many commercial PBPK platforms [118]. A detailed overview on PBPK model constructions is reported, along with examples of in silico simulations successfully establishing high IVIVCs for furosemide, fenofibrate, rifampicin in LBFs [119, 120].

A mechanistic PBPK model was built for the current commercial formulation of VTX by Rigemaier et al. [7]. Inputs for this model included physiochemical properties of the drug, solubility/permeability of VTX, dissolution data from the marketed amorphous solid dispersion formulation, pharmacokinetic parameters and metabolic pathways [7]. The model was verified against studies assessing drug-drug interactions, accurately predicting the effect of a CYP3A inhibitor and inducer on plasma VTX concentrations [7]. Given this, the model can further be applied for assessing untested VTX-drug interactions of mild and intermediate modulators of CYP3A in healthy volunteers [7]. The existing PBPK model for Venclexta^®^ is based on an amorphous solid dispersion and has not yet been adapted or validated for novel LBFs of Venetoclax. However, PBPK models developed for LBFs of other drugs indicate that accurate model predictions require experimentally measured LBF-specific data as inputs. This includes integrating the biorelevant apparent solubility of the drug in the LBF, biorelevant dissolution profiles, in vitro and *ex vivo* permeation profiles, as well as formulation particle morphology and droplet size distributions [121]. Henze et al. tested the commercial tablets of VTX in biorelevant fasted state porcine media, and in combination with PBPK modelling, concluded that using porcine media resulted in reliable predictions of plasma concentration profiles in fasted pigs [122]. Although still a relatively underexplored area of research, such findings underline the value predictive power of these tools.

## Conclusion

Addressing current delivery and formulation challenges of VTX to a greater extent can provide patients with greater drug absorption at a lower burden of dose, consumption of pills and removal of the need of food consumption. This review discussed previously applied bio-enabling strategies, namely amorphous solid dispersions, nanocrystals, liquid SEDDS, one supersaturated liquid LBFs with and without precipitation inhibitors, and incorporation of a lipophilic salt into a liquid SEDDS, on enhancing the in vitro solubilisation and in vivo absorption properties of VTX. In comparison to the extensive work conducted with LBFs for other PWSDs within the literature, minimal work has been conducted with VTX despite its high clinical relevance. A novel fabrication approach that combines LBF application, solidification, and supersaturation is relatively underexplored and shows promising signs for application to VTX with downstream enhanced drug release, improved oral absorption and bioavailability, and a lower amount of daily drug dose. Application of heat-induced supersaturation to lipophilic salts can achieve higher drug solubility compared to a supersaturated version of pure drug counterparts. Inorganic materials such as polymers and mesoporous silica also remains largely untrialled for VTX, as a BCS Class IV. Each of these formulation strategies will require rational section of components, whether they are lipid-based, silica or polymer-based, in addition to the appropriate supersaturation technique and solid carrier for specific formulations, and in vivo evaluation to assess absorption characteristics. Notably, none of the existing studies have investigated biodistribution of VTX into various organs, signalling a clear knowledge gap on the efficacy-toxicity relationship. Critical study design adjustments such as comparing innovative formulations with the current commercial formulation during pharmacokinetic assessments can equip formulators with comprehensive understanding of formulation behaviour and success. Nevertheless, limited clinical translation of LBFs persists, largely due to the inadequacy of current in vitro and preclinical in vivo studies to dictate the performance of such systems. Incorporating physiologically based pharmacokinetics modelling can equip formulators to understand the fate of their systems directly in a human model, minimizing the need for animal testing where correlation and complexity is low. Ultimately, the integration of advanced formulation sciences, pharmacometrics modelling, clinical expertise and pharmacokinetic/pharmacodynamic studies is crucial to enabling the development and clinical translation of bio-enabling strategies for the optimal use of VTX in patients.

## Data Availability

No datasets were generated or analysed during the current study.
